# A Body Shape Index shows Stronger Association with Falls than BMI in Older Adults

**DOI:** 10.1093/geroni/igaf122.3687

**Published:** 2025-12-31

**Authors:** Zhenxuan Ma, Indrakshi Roy

**Affiliations:** Indiana University at Bloomington, Bloomington, Indiana, United States; Indiana University, Bloomington, Bloomington, Indiana, United States

## Abstract

Falls are a major public health concern among older adults, frequently leading to severe injuries, loss of independence, and increased mortality. Identifying individuals at higher risk of falls is therefore a priority in geriatric care and preventive strategies. While Body Mass Index (BMI) has traditionally been used to assess body composition, it fails to account for fat distribution and central obesity, which critically influence postural stability and fall risk. A Body Shape Index (ABSI), a novel anthropometric measure incorporating waist circumference with height and weight, has been proposed as a more accurate indicator of abdominal adiposity. This study aimed to compare the association of ABSI versus BMI in relation to falls among older adults, utilizing data from the National Health and Aging Trends Study (NHATS). This retrospective cohort study analyzed data from 5,343 NHATS participants aged 65 years and older. In adjusted models, ABSI was associated with both any fall (OR 1.15, 95% CI 1.04–1.27, p = 0.009) and multiple falls (OR 1.20, 95% CI 1.03–1.39, p = 0.018), while BMI showed weaker associations (any fall: OR 1.02, 95% CI 1.00–1.03, p = 0.030; multiple falls: OR 1.01, 95% CI 0.995–1.03, p = 0.158). Associations were stronger among adults ≥80 years, those with multiple chronic conditions (MCC), and in subgroups with specific diseases such as arthritis, hypertension, or diabetes. This study suggests ABSI may be a more sensitive measure than BMI for identifying fall risk and may provide added value for geriatric fall risk assessment.

